# Characterization of the Novel Leaderless Bacteriocin, Bawcin, from *Bacillus wiedmannii*

**DOI:** 10.3390/ijms242316965

**Published:** 2023-11-30

**Authors:** Zafina Budhwani, Jenna T. Buragina, Jen Lang, Jeella Z. Acedo

**Affiliations:** 1 Department of Chemistry and Physics, Mount Royal University, Calgary, AB T3E 6K6, Canada; zafina.budhwani@mail.mcgill.ca; 2 Department of Biology, Mount Royal University, Calgary, AB T3E 6K6, Canada; jenna.buragina@ucalgary.ca (J.T.B.);

**Keywords:** leaderless bacteriocin, antimicrobial, antimicrobial resistance

## Abstract

The rise of drug-resistant bacteria is a major threat to public health, highlighting the urgent need for new antimicrobial compounds and treatments. Bacteriocins, which are ribosomally synthesized antimicrobial peptides produced by bacteria, hold promise as alternatives to conventional antibiotics. In this study, we identified and characterized a novel leaderless bacteriocin, bawcin, the first bacteriocin to be characterized from a *Bacillus wiedmannii* species. Chemically synthesized and purified bawcin was shown to be active against a broad range of Gram-positive bacteria, including foodborne pathogens *Staphylococcus aureus*, *Bacillus cereus*, and *Listeria monocytogenes*. Stability screening revealed that bawcin is stable over a wide range of pH (2.0–10.0), temperature conditions (25–100 °C), and against the proteases, papain and pepsin. Lastly, three-dimensional structure homology modeling suggests that bawcin contains a saposin-fold with amphipathic helices and a highly cationic surface that may be critical for membrane interaction and the subsequent cell death of its targets. This study provides the foundational understanding of the activity and properties of bawcin, offering valuable insights into its applications across different antimicrobial uses, including as a natural preservative in food and livestock industries.

## 1. Introduction

The discovery of antibiotics has saved many lives from infections for decades. Yet, the continuous emergence of antimicrobial resistance (AMR) is leaving traditional antibiotics ineffective. This evolution has given rise to various multidrug-resistant strains that significantly threaten human health. The World Health Organization has recognized AMR as one of the top 10 global public health concerns (https://www.who.int/news-room/fact-sheets/detail/antimicrobial-resistance; accessed 11 October 2023), where 1.27 million deaths in 2019 were related to bacterial AMR alone [1]. Hence, the scientific community has been exploring several avenues to combat AMR, including the discovery of antimicrobial alternatives such as bacteriocins [2]. Bacteriocins are bacteria-derived ribosomally synthesized antimicrobial peptides that are active against other bacteria, either in the same species (narrow spectrum) or across genera (broad spectrum) [3]. These compounds are particularly attractive alternatives to antibiotics because they are natural, can be genetically modified, and are normally highly stable and non-cytotoxic [4].

Bacteriocins can be grouped based on their general structure and biology; however, multiple classification schemes have been proposed and continue to change as more information about bacteriocins is discovered. The most current proposed classification includes three major classes that are distinguished by heat stability, post-translational modifications, and size [5]. Each class can be further subdivided according to other structural features, such as “leaderless” bacteriocins within class II, and specifically classified as class IId [5]. Leaderless bacteriocins are of particular interest because they are not produced with a leader peptide. Bacteriocins are normally synthesized as inactive precursor peptides consisting of an N-terminal leader peptide and a C-terminal core peptide, where the leader sequence must be cleaved before the active bacteriocin is released [6]. Leaderless bacteriocins, however, are active upon translation and do not contain a leader sequence [3]. The leader peptide is known to play an essential role in bacteriocin biosynthesis such as in conferring producer organism immunity and serving as recognition motifs for biosynthetic enzymes [6,7]. The absence of a leader peptide in leaderless bacteriocins therefore renders their immunity and transport mechanisms unconventional and enigmatic [6,7].

Bacteriocin production is a widespread feature of bacterial species [8,9]; however, identifying bacteriocins from *Bacillus* species is of particular interest given their high capacity for antimicrobial compound production [10]. *Bacillus* bacteria can produce a wide variety of bacteriocins spanning nearly all bacteriocin classes [10], and new species of this genus continue to be discovered [11]. Here, we investigated the activity and stability of a novel leaderless bacteriocin termed bawcin, which is the first bacteriocin to be characterized from the recently established *Bacillus wiedmannii* species [11].

## 2. Results and Discussion

### 2.1. Bawcin Identification and Biosynthetic Gene Cluster

**Figure 1:**
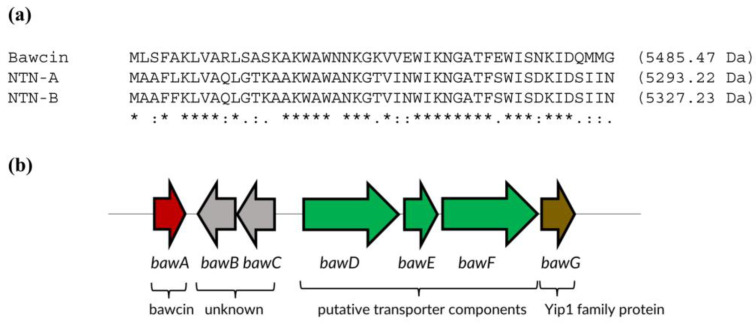
(**a**) Amino acid sequence alignment of bawcin, NTN-A, and NTN-B. Sequences were aligned using Clustal Omega [18]. Conserved, conservative, and semi-conservative substitutions are indicated by asterisks, colons, and periods, respectively. (**b**) Biosynthetic gene cluster of bawcin (BawA). The red arrow refers to the bacteriocin structural gene (*bawA*); gray arrows are genes (*bawB* and *bawC*) encoding hypothetical proteins of unknown function; green arrows represent genes encoding putative transporter proteins, specifically, *bawD* (HlyD family efflux transporter), *bawE* (ABC transporter ATP-binding protein), and *bawF* (ABC transporter permease); and the brown arrow indicates the *bawG* gene encoding a Yip1 family protein.

Our recent genome mining study identified over 400 producer organisms that putatively produce novel leaderless bacteriocins [12]. In this earlier work, an all-by-all BLAST analysis was performed to compare the amino acid sequences of characterized and putative leaderless bacteriocins. Results were visualized through a sequence similarity network wherein the peptides were grouped based on sequence similarity. Bawcin belongs to Group 1, which is the largest among the 11 groups identified. In detail, Group 1 includes 76 different bacterial strains, mostly *Bacillus* species, with a few *Oceanobacillus, Parageobacillus*, and *Virgibacillus* strains. When we first identified bawcin as a candidate peptide to isolate and characterize, there were no known members of the said group, and we aimed to report the first representative member of this family. However, more recently, the bacteriocins NTN-A and NTN-B from *Virgibacillus salexigens* NT N53, isolated from the deep-sea floor, were identified and found to belong to the same family as that of bawcin [13]. NTN-A and NTN-B are 98% identical, while bawcin displays 62.5% sequence identity with NTN-A and NTN-B (Figure 1a). In contrast to the discovery of NTN-A and NTN-B, which were isolated from their natural producer organism, we identified bawcin in silico, and it was synthesized chemically through solid-phase peptide synthesis and HPLC-purified for subsequent characterization. Its identity was confirmed by mass spectrometry.

*B. wiedmannii* was recently established as a novel species through an in-depth characterization of 11 *B. wiedmannii* sp. nov strains, all found in dairy environments between 2005 and 2012 [11]. This species is represented by the type strain FSL W8-0169^T^, which was isolated in 2012 from an American dairy powder processing plant raw milk silo [11,14]. Bawcin represents the first bacteriocin to be identified from a *B. wiedmannii* species. It is specifically encoded in the genomes of *B. wiedmannii* strains AFS001911 and AFS057349, which were isolated from a plant core by AgBiome (Durham, NC, USA) [15,16].

Bawcin was found to be a water-soluble peptide that has 48 amino acids and a molecular weight of 5485.47 Da (Figure 1a; protein accession number WP_098048139.1). Its biosynthetic gene cluster (BGC) (Figure 1b) has seven genes, four of which are putative membrane proteins (Table 1). Downstream the bawcin structural gene (*bawA*) are two genes (*bawB* and *bawC*) that encode proteins with unknown function. BawB is a hypothetical protein with 94 amino acid residues and three predicted transmembrane domains. BawC is a soluble protein with 98 residues. BawDEF are putatively involved in bawcin transport and secretion and consist of 390, 226, and 399 residues, respectively. In particular, BawD is a putative HlyD family efflux transporter with one transmembrane domain, BawE is a putative soluble ATP-binding ABC transporter, and BawF is a putative ABC transporter permease with five membrane-spanning domains. Lastly, BawG is a Yip1 family membrane protein with 223 residues and five predicted transmembrane domains. Yip1 proteins are postulated to be involved in transport and were identified to be prevalent in BGCs of circular bacteriocins that specifically involve the set of three genes encoding ABC transporter components, similar to the ones present in the bawcin BGC (i.e., *bawDEF*) [17].

### 2.2. Antimicrobial Activity of Bawcin

Bawcin is active against nine of the fourteen bacterial species tested (Table 2). Given bawcin inhibited the growth of various bacteria outside the *Bacillus* genus, it is considered a broad-spectrum bacteriocin. Its activity against multiple notable foodborne pathogens, including *S. aureus*, *B. cereus*, and *L. monocytogenes* [20], demonstrates bawcin’s potential as a promising food biopreservative. In addition, antimicrobials with a broad activity range are useful for the treatment of infections in which the causative bacterial pathogen is unknown. However, they may negatively impact the human microbiome [21]. Bioengineering [22] and combinatorial therapy [23] can enhance the potency of bacteriocins to target species and therefore be used to reduce the impact of broad-spectrum bacteriocins on host microbiota.

The minimum inhibitory concentrations (MICs) of bawcin are comparable to those of other broad-spectrum leaderless bacteriocins, specifically those peptides that were tested using an agar-based assay such as the spot-on-lawn assay employed in this study [24,25,26]. The spot-on-lawn assay allows for the detection of bacterial heteroresistance (HR). HR is the phenomenon in which a portion of cells within an otherwise susceptible bacterial population is resistant to a given antimicrobial [27,28]. HR is distinct from persistence, given the cells are able to proliferate in the presence of the antimicrobial [29]. Since heteroresistant populations can grow in the presence of antibiotics, understanding and controlling HR is necessary to avoid infection treatment failure [27,28]. Bawcin HR was observed for all susceptible species, although at a minimal level for most organisms (Table 2). In order to effectively utilize bawcin against these species, techniques to avoid HR should be pursued in future studies such as through combined application with other antimicrobials that could enhance bawcin’s activity [30,31].

*S. epidermidis*, *S. pyogenes*, *G. stearothermophilus*, *P. aeruginosa*, and *S. enterica* were unaffected by bawcin at all concentrations (Table 2). A lack of activity against *P. aeruginosa* and *S. enterica* was expected given Gram-negative species are typically resistant to bacteriocins due to their reduced cell wall permeability [3]. Inactivity against these two organisms is consistent with leaderless bacteriocins produced by other *Bacillus* species that have been shown to be bactericidal via inducing bacterial membrane damage [32,33,34]. Given their comparable antimicrobial spectra, bawcin may exhibit antimicrobial activity through a similar mechanism. Further, resistance by the Gram-positive species, *S. epidermidis*, *S. pyogenes*, and *G. stearothermophilus*, may be due to structural differences, such as a lack of a receptor necessary for peptide docking to the membrane. Generally, bacteriocins of this class are believed to initiate pore formation without target receptors [35] and can even exhibit bactericidal effects without generating membrane pores in target bacteria [36]. However, there are a few leaderless bacteriocins that use receptors, such as LsbB, which only inhibits the growth of bacteria expressing the YvjB Zn-dependent metallopeptidase [37]. Factors contributing to the need for bacteriocin receptors are poorly understood [38]; yet, the specificity of bawcin to only certain Gram-positive species suggests a receptor may be necessary for its antimicrobial activity. Evidently, additional studies are necessary to establish bawcin’s mode of action and potential target receptor, which will provide insights into what contributes to bawcin’s selectivity and the additional techniques that are best suited to enhance its potency and antimicrobial spectrum.

### 2.3. pH and Temperature Stability of Bawcin

**Figure 2:**
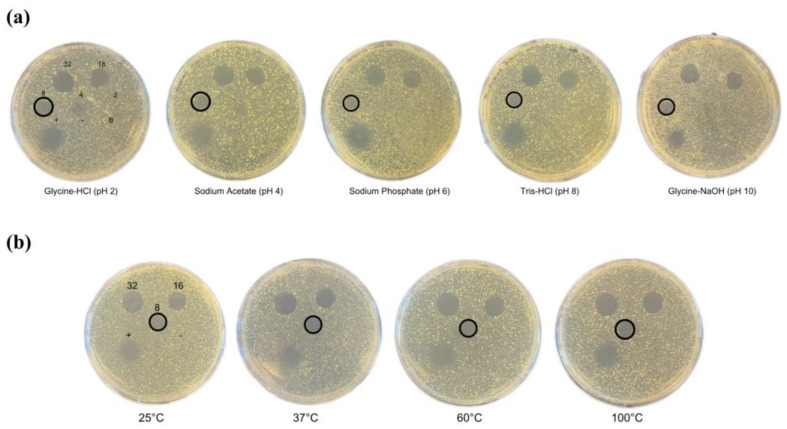
Representative spot-on-lawn assay results for bawcin at various (**a**) pH values (pH 2.0–10.0) and (**b**) temperatures (25–100 °C) against *M. luteus* after 1 h of incubation. Bawcin retained its activity at 8 µM across all conditions (circled). Respective ampicillin (positive control, +), sterile water (negative control, -), and buffer (B) controls were also spotted. Assays were performed in triplicates.

Understanding bawcin’s stability and activity in various chemical and temperature environments is important as this information will guide the use of bawcin for various practical and industrial applications. Bawcin remained active at the MIC (8 µM) without heteroresistance development after exposure to pH 2.0–10.0 against *M. luteus* (Figure 2a). This activity can be attributed only to bawcin and not from any adverse buffer effects against the indicator organism, as the buffer control did not produce a zone of inhibition. The incubation time for the pH testing procedure was optimized such that the bacteriocin-buffer solutions were incubated for 30, 60, and 120 min. At 30 min after incubation, the peptide did not appear to have fully equilibrated with the buffer. The 60 and 120 min incubation times produced consistent results; therefore, 60 min was selected for the assay. Similarly, bawcin also remained active against *M. luteus* after 1 h exposure to 25–100 °C (Figure 2b), independent of the pH results.

The *Bacillus* genus has been heavily studied with respect to the bacteriocins and bacteriocin-like inhibitory substances they produce [10,39,40]. This is because this genus has been implicated in various industrial processes, is generally regarded as safe, easy, and inexpensive to grow; and tends to have broad-range inhibitory effects [10]. Similar to the stability testing results we obtained for bawcin, several independent studies of bacteriocins from *Bacillus* species have demonstrated that these peptides are stable across a wide pH and temperature range [41,42,43].

Industrial applications of bacteriocins are currently expanding. For example, nisin was first commercially introduced in the 1950s as a food preservative in the UK [44,45]. Recently, bacteriocins are also being considered for use in the cosmetic industry and human and veterinary medicine [46]. Additionally, there are efforts to improve bacteriocins’ antimicrobial efficacy in various industrial applications such as using encapsulation, bioengineering, combinatory/complementary methods, and creating active antimicrobial packaging [47]. With the growing interest in bacteriocin industrial applications, bawcin has the potential to be used for diverse applications due to its thermal and chemical stability, and further research can be directed towards optimizing conditions for its use in specific applications.

### 2.4. Protease Stability of Bawcin

**Figure 3:**
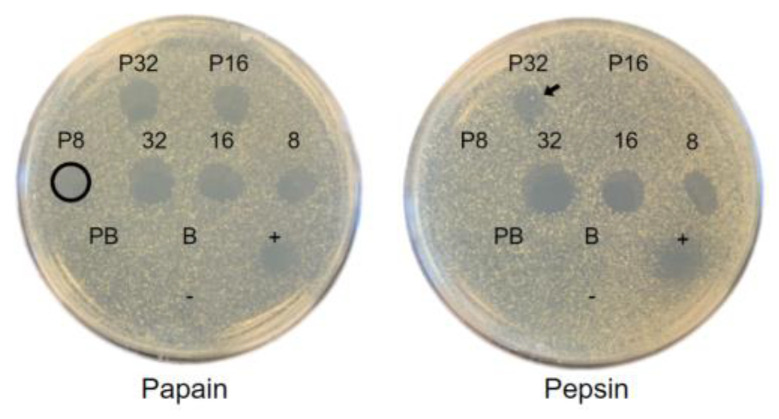
Representative spot-on-lawn assay results for bawcin at 32, 16, and 8 µM against *M. luteus* following exposure to 1 mg/mL papain and pepsin for 1 h at 37 °C. Bawcin remained active against *M. luteus* at 8 µM in combination with papain (circle) and partial activity at 32 µM with pepsin (arrow). Ampicillin (positive control, +), sterile water (negative control, -), protease (P), and buffer (B) controls were also spotted. Assays were performed in triplicates.

Bawcin retained its antimicrobial activity at 1×, 2×, and 4× the MIC when combined with papain and only at 4× the MIC (with HR) with pepsin (Figure 3). Bawcin lost antimicrobial activity at all concentrations tested in the presence of trypsin, alpha-chymotrypsin, and proteinase K. Bawcin’s altered antimicrobial activity in combination with proteases confirms its proteinaceous nature and highlights its potential for food preservation. Given antibiotics have been historically overused in food production [48] and there is an increasing demand for naturally derived additives, developing bacteriocins as food preservatives has become a popular area of research. To qualify as a food additive, a bacteriocin must (a) be non-toxic to human cells and gut microbiota; (b) impair the growth of food contaminant bacteria; (c) retain activity in the target food matrix; (d) and have high stability in different acidity, temperature, and salt levels [49]. Inactivation of bawcin by the digestive proteases, pepsin, trypsin, and alpha-chymotrypsin, demonstrates that bawcin will likely be safely degraded within the human gastrointestinal tract upon consumption. In addition to several foodborne pathogens [20], beneficial bacteria, including *L. casei*, *L. delbruekii* ssp. *bulgaricus*, and *B. subtilis*, are sensitive to bawcin (Table 2). Yet, nisin is also active against these species, has been FDA-approved, and is generally recognized as safe [50]. To understand bawcin’s potential in food preservation, future efforts should focus on further establishing bawcin’s toxicity and stability in food matrices. Although bawcin is resistant to papain (Figure 3), a protease commonly found in meat as a tenderizer [51], other considerations beyond the presence of proteolytic enzymes include the degree in which bawcin may interact with food components and experience changes in solubility and charge [52].

Despite inactivation within the digestive system being advantageous with respect to food applications, it is a barrier to developing bawcin for orally administered pharmaceutical use against bacterial infections. However, this issue may be resolved as nanoencapsulation delivery systems become more advanced [53] or through genetic modification [54]. For instance, inserting ᴅ-amino acid residues within peptide terminal ends has been shown to reduce protease sensitivity [55,56], and this technique resulted in a relatively small loss of antimicrobial activity when implemented in bacteriocins previously [54].

### 2.5. Structural Analysis of Bawcin

**Figure 4:**
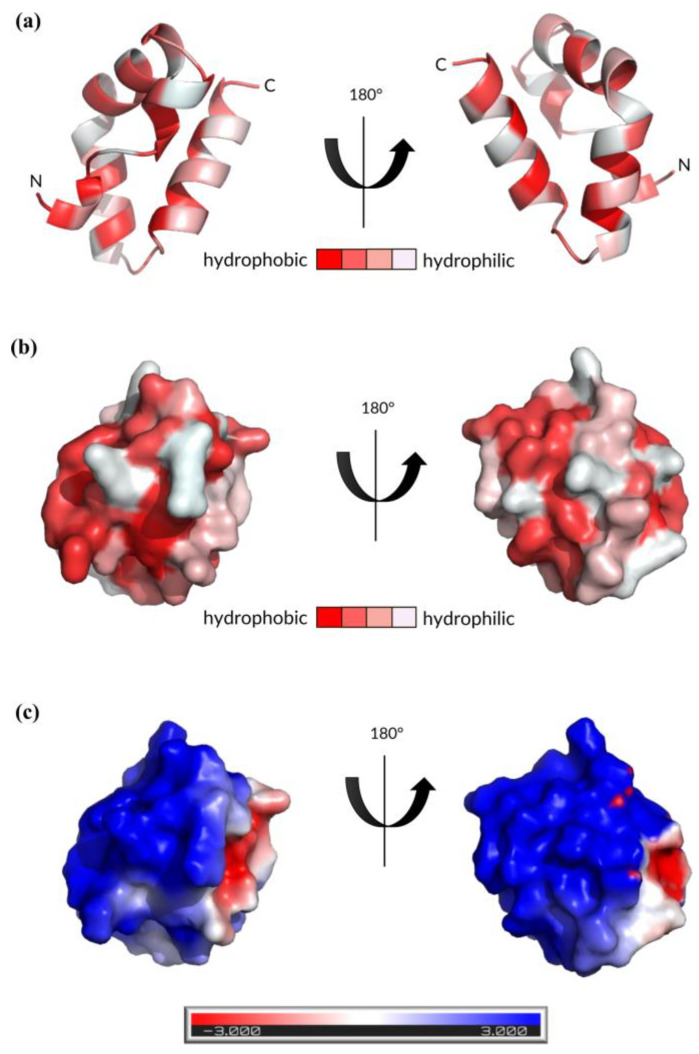
(**a**) Predicted three-dimensional structure of bawcin obtained using the SWISS-MODEL tool [57] and visualized using PyMOL [58]. The N- and C- termini are labelled. A gradient color scheme is used where darker red color indicates increased hydrophobicity. (**b**) Hydrophobicity surface map of bawcin generated using PyMOL. (**c**) Electrostatic potential surface maps of bawcin generated using APBS functionality of the PDB2PQR online pipeline [59]. Cationic regions are colored blue.

The three-dimensional structure of bawcin was predicted using the SWISS-MODEL server [57], visualized using PyMOL 2.0 [58], and shown to consist of four amphipathic alpha-helices (Figure 4a). In detail, each helix has a distinct strip of hydrophobic residues that are oriented toward the core of the structure, while a hydrophilic strip is exposed on the surface.

The hydrophobicity surface maps reveal hydrophobic patches on the protein’s surface (Figure 4b). These structural features of bawcin are characteristic of the saposin-like fold structure adopted by many leaderless bacteriocins [60]. Saposin-like folds rely on a combination of hydrophobic and electrostatic interactions to stabilize the structure of the peptide [3]. Although bawcin presents exposed hydrophobic patches, the peptide remains hydrophilic in character as it is fully soluble in water and has a high isoelectric point of 10.12. Moreover, the electrostatic surface maps reveal bawcin has a net cationic surface (Figure 4c). Specifically, the primary sequence of bawcin has eight cationic residues (i.e., seven lysine and one arginine). The hydrophobic and electrostatic characteristics of bawcin, like with other leaderless bacteriocins, are thought to play a key role in the bacteriocin’s mode of action, particularly in anchoring and attracting a bacterial cell membrane [61]. Further investigation into bawcin would be required to determine its specific mode of action.

## 3. Materials and Methods

### 3.1. Bawcin and Bacterial Strains

Bawcin was chemically synthesized and HPLC-purified by Biomatik, consistent with its predicted amino acid sequence (MLSFAKLVARLSASKAKWAWNNKGKVVEWIKNGATFEWISNKIDQMMG) and as confirmed by mass spectrometry. *B. cereus* (Ward’s 85W 0200), *B. subtilis* (Ward’s 85W 1650), *E. faecalis* (Ward’s 85W 1100), *L. casei* (Ward’s 85W 1682), *L. lactis ssp. lactis* (Ward’s 85W 1774), *L. monocytogenes* ATCC 19115, *M. luteus* (Ward’s 85W 0966), *S. aureus* ATCC 6538, *S. epidermidis* (Ward’s 85W 1747), *S. pyogenes* (Ward’s 85W 1180), *L. delbruekii ssp. bulgaricus* (VWR 470179-126), *G. stearothermophilus* 1010, *P. aeruginosa* (Ward’s 85W 1173), and *S. enterica* (Ward’s 85W 1710) were used in this study.

### 3.2. Antimicrobial Activity Testing

The MIC of bawcin against each bacterial strain was determined using the spot-on-lawn assay as described previously with modifications [62]. A Mueller Hinton (MH) double-agar plate was prepared by pouring 5 mL of 0.75% MH agar inoculated with 50 µL of 0.10 OD_625_ bacterial suspension onto 20 mL of dried 1.5% MH agar. Bawcin diluted with sterile water to 128, 64, 32, 16, 8, 4, 2, and 1 µM was spotted in 10 µL aliquots onto the top agar layer, along with 10 µL of an antibiotic positive control (ampicillin or kanamycin) and sterile water negative control. Plates were incubated at 37 °C for 16–20 h and the MIC was identified as the lowest concentration in which a zone of inhibition was observed, where partial bacterial growth within the zone of inhibition was considered HR. Assays were performed in triplicate.

### 3.3. pH Stability Testing

pH screening methods were performed as described previously with modifications [62,63]. The following 100 mM buffer solutions were prepared: glycine-HCl (pH 2), sodium acetate (pH 4), sodium phosphate (pH 6), Tris-HCl (pH 8), and glycine NaOH (pH 10). Bacteriocin-buffer solutions at various pH values and concentrations were prepared by combining equal volumes of buffer and peptide solutions. The final concentrations of buffer solutions and bawcin were 50 mM and half the original bawcin concentration, respectively. To examine pH stability, bacteriocin-buffer solutions were spotted according to the spot-on-lawn assay described above following 1 h of incubation at room temperature. *M. luteus* was used as the indicator strain, with ampicillin, sterile water, and buffer spotted as positive, negative, and blank controls, respectively. Assays were performed in triplicate.

### 3.4. Temperature Stability Testing

The thermal stability of bawcin was examined by incubating aliquots of desired bawcin concentrations at 25, 37, 60, and 100 °C for 1 h. Residual activity was then measured using the spot-on-lawn assay method with *M. luteus* as the indicator strain as described above with ampicillin and sterile water spotted as positive and negative controls, respectively. Assays were performed in triplicate.

### 3.5. Protease Sensitivity Testing

Protease stability testing was evaluated as described previously with modification [64]. Papain (Scholar Chemistry, Rochester, NY, USA), pepsin (Northwest Scientific, Billings, MT, USA), trypsin (Sigma-Aldrich, Burlington, MA, USA), alpha-chymotrypsin (Worthington, Lakewood, NJ, USA), and proteinase K (Fisher Scientific, Ottawa, ON, Canada) were prepared to a final concentration of 1 mg/mL in 50 mM phosphate buffer adjusted to pH 2 (pepsin), 6 (papain), and 8 (proteinase K, trypsin, alpha-chymotrypsin). Bawcin was combined with each protease to final concentrations of 1×, 2×, and 4× the MIC. Bawcin’s residual antimicrobial activity against *M. luteus* was determined via spot-on-lawn assay as described above. The following samples were spotted for each assay: (1) protease combined with bawcin at each concentration, bawcin alone at each concentration, buffer alone, protease in buffer alone, ampicillin (positive control), and sterile water (negative control), where all solutions were incubated for 1 h at 37 °C prior to spotting except ampicillin and sterile water. Assays were performed in triplicate.

### 3.6. Sequence and Structural Analysis

The three-dimensional structure of bawcin was predicted using SWISS-MODEL [57] and visualized using PyMOL [58]. A hydrophobic surface map was created using PyMOL and an electrostatic potential map was generated using the APBS extension with the PDB2PQR molecule preparation method. Primary sequence alignments were performed using Clustal Omega [18], and sequence parameters were calculated using ExPASy ProtParam [65]. The number of putative transmembrane domains was deduced using the SOSUI program [19].

## 4. Conclusions

The present study identified, characterized, and evaluated the novel bacteriocin, bawcin, the first bacteriocin to be characterized from a *B. wiedmannii* species. Bawcin was shown to be a broad-spectrum bacteriocin with considerable stability against pH and temperature changes, as well as the proteolytic activity of papain and pepsin. Three-dimensional structure homology modeling suggests that bawcin adopts a saposin-like fold with a highly cationic surface that could facilitate its binding to target cell membranes, leading to subsequent cell death. Given bawcin’s activity and remarkable stability, it has evident potential for diverse industrial applications, including within the food industry, as an alternative or additive to existing antimicrobials. Future studies can be directed toward identifying suitable industrial contexts and optimal conditions for bawcin’s application, as well as elucidating bawcin’s mode of action.

## Figures and Tables

**Table 1:** Predicted proteins encoded by the bawcin biosynthetic gene cluster ^1^.

Protein	Size (aa)	TM	Identity
BawA	48	0	Bawcin (bacteriocin)
BawB	94	3	Hypothetical protein
BawC	98	0	DUF2089 family protein
BawD	390	1	HlyD family efflux transporter
BawE	226	0	ABC transporter ATP-binding protein
BawF	399	5	ABC transporter permease
BawG	223	5	Yip1 family protein

^1^ aa, amino acids; TM, transmembrane domains as predicted using the SOSUI program [19].

**Table 2:** Minimum inhibitory concentration (MIC) of bawcin against tested bacteria using spot-on-lawn assay ^1^.

Indicator Organism	MIC (µM)	Heteroresistance (µM)
*Micrococcus luteus* (Ward’s 85W 0966)	4	4
*Lactococcus lactis* ssp. *lactis* (Ward’s 85W 1774)	4	4
*Lactobacillus delbruekii* ssp. *bulgaricus* (VWR 470179-126)	8	8
*Bacillus subtilis* (Ward’s 85W 1650)	8	8, 16
*Listeria monocytogenes* ATCC 19115	8	8, 16
*Lactobacillus casei* (Ward’s 85W 1682)	8	8–32
*Bacillus cereus* (Ward’s 85W 0200)	16	16–64
*Staphylococcus aureus* ATCC 6538	16	16–64
*Enterococcus faecalis* (Ward’s 85W 1100)	32	32–128
*Staphylococcus epidermidis* (Ward’s 85W 1747)	-	-
*Streptococcus pyogenes* (Ward’s 85W 1180)	-	-
*Geobacillus stearothermophilus* 1010	-	-
*Pseudomonas aeruginosa* (Ward’s 85W 1173)	-	-
*Salmonella enterica* (Ward’s 85W 1710)	-	-

^1^ -, no activity.

## Data Availability

The authors confirm all supporting data, code and protocols have been provided within the article. The accession number for bawcin is WP_098048139.1 and can be accessed here: https://www.ncbi.nlm.nih.gov/protein/WP_098048139.1; accessed 10 January 2023.
